# SRSF3 functions as an oncogene in colorectal cancer by regulating the expression of ArhGAP30

**DOI:** 10.1186/s12935-020-01201-2

**Published:** 2020-04-10

**Authors:** Ji-Lin Wang, Chun-Rong Guo, Tian-Tian Sun, Wen-Yu Su, Qiang Hu, Fang-Fang Guo, Lun-Xi Liang, Jie Xu, Hua Xiong, Jing-Yuan Fang

**Affiliations:** 1 Division of Gastroenterology and Hepatology, Key Laboratory of Gastroenterology and Hepatology, Ministry of Health, State Key Laboratory for Oncogenes and Related Genes, Renji Hospital, School of Medicine, Shanghai Institute of Digestive Disease, Shanghai Jiao Tong University, 145 Middle Shandong Road, Shanghai, 200001 China; 2 The Centre of Teaching and Experiment, Shanghai University of Traditional Chinese Medicine, Pudong District, Shanghai, China

**Keywords:** SRSF3, ArhGAP30, Colorectal cancer

## Abstract

**Background:**

Splicing factor SRSF3 is an oncogene and overexpressed in various kinds of cancers, however, the function and mechanism involved in colorectal cancer (CRC) remained unclear. The aim of this study was to explore the relationship between SRSF3 and carcinogenesis and progression of CRC.

**Methods:**

The expression of SRSF3 in CRC tissues was detected by immunohistochemistry. The proliferation and invasion rate was analyzed by CCK-8 assay, colony formation assay, transwell invasion assay and xenograft experiment. The expression of selected genes was detected by western blot or real time PCR.

**Results:**

SRSF3 is overexpressed in CRC tissues and its high expression was associated with CRC differentiation, lymph node invasion and AJCC stage. Upregulation of SRSF3 was also associated with shorter overall survival. Knockdown of SRSF3 in CRC cells activated ArhGAP30/Ace-p53 and decreased cell proliferation, migration and survival; while ectopic expression of SRSF3 attenuated ArhGAP30/Ace-p53 and increases cell proliferation, migration and survival. Targeting SRSF3 in xenograft tumors suppressed tumor progression in vivo.

**Conclusions:**

Taken together, our data identify SRSF3 as a regulator for ArhGAP30/Ace-p53 in CRC, and highlight potential prognostic and therapeutic significance of SRSF3 in CRC.

## Background

Colorectal cancer (CRC) is one of the most commonly diagnosed cancer in the world, with over one million new cases every year in the world [1]. CRC usually arises through a multistep process, including multiple genetic and epigenetic alterations that drive malignant transformation. However, the detailed molecular mechanisms involved in CRC have not yet been fully characterized.

SR proteins (serine/arginine-rich proteins), functioning as mRNA-binding proteins and alternative splicing factors, have diverse cellular functions including transcription, translation, RNA export, genomic stability, and miRNA processing [2]. Since SR proteins participate in various cellular processes, their aberrant expression may cause various diseases including cancers [3]. SRSF3 (serine/arginine-rich splicing factor 3), also known as SRp20, is one of the most famous SR proteins. SRSF3 is a multi-functional protein, and has been found to be involved in various human diseases, including cancers [4]. It is reported that SRSF3 is frequently overexpressed in most types of cancer and implicated in poor prognosis of cancer patients [5–8]. SRSF3 is also involved in colorectal carcinogenesis [9, 10], however, the specific expression patten and mechanism of SRSF3 in CRC is still unclear.

In our previous study, we found that ArhGAP30 (Rho GTPase activating protein 30) functioned as a tumor-suppressor gene in CRC by promoting the acetylation and functional activation of p53 [11], however, the mechanism of ArhGAP30 inactivation in CRC remained unknown. In this study, we found that SRSF3 could suppress the expression and function of ArhGAP30. We also found SRSF3 protein level was significantly unregulated in CRC tissues. Overexpression of SRSF3 could increase the proliferation and invasion of CRC in vitro and in vivo. By these approaches we aim to elucidate the role of SRSF3 in the regulation of ArhGAP30 and evaluate SRSF3 as a potential biomarker for the prognosis CRC.

## Materials and methods

### Patients and CRC biopsy specimens

A total of 20 pathologically confirmed CRC patients were enrolled and underwent surgery at Ren-ji Hospital, affiliated to the Shanghai Jiao-tong University School of Medicine, between January 2016 and May 2016. The study was approved by the ethics committee of Shanghai Jiao-tong University School of Medicine (No. 81502015), and written informed consent was obtained from all patients at study entry. One tissue microarray including 90 pairs of CRC and corresponding non-tumor tissues were purchased from BioChip (Shanghai, China). For the detailed characteristics of the 90 CRC cases, 47 was male, 43 was female; 28 were younger than 60, while 62 older than 60; 44 located in the left colon and rectum, 46 located in the right colon; the tumor size of 39 cases were smaller than 5 cm, while 51 larger than 5 cm; for differentiation, 76 were classified as grade 1–2, and 14 as grade 3–4; for T stage, 13 was grade 1–2, and 77 grade 3–4; 59 cases had no lymph node invasion, 31 had one or more lymph node invasion; 87 cases had no metastases, while 3 cases had distance metastases; for AJCC stage, 57 was classified as grade 1–2, and 33 as grade 3–4; All of the CRC tissues were confirmed as adenocarcinoma. However, only one core from each tumor was included in the TMA, therefore, tumor heterogeneity may exist in this study just as most of other studies using TMA.

### Immunohistochemistry analysis of SRSF3

Methods for tissue immunohistochemistry (IHC) have been described previously [12]. In short, the protein expression was examined using a two-step streptavidin–biotinperoxidase method with SRSF3 primary antibody. The antigen retrieval was conducted by microwave irradiation. Staining was performed using the DAB kit (Maixin Bio, Shanghai, China), according to the manufacturer’s instructions. The slides were independently examined by two investigators blinded to both the clinical data and pathology. PBS replaced SRSF3 primary antibody was used as a negative control. Esophageal small-cell carcinoma tissue was used as a positive control because almost all of the esophageal small-cell carcinoma cells show strong staining for SRSF3. The protein expression of SRSF3 was firstly examined in 20 CRC tissues and the paired normal tissues using IHC. Then, it was confirmed in a tissue microarray including 90 pairs of CRC and corresponding non-tumour tissues.

The primary antibody was purchased from Abcam, diluted at 1:100.

### Immunofluorescence

The tissue sections were deparaffinized in xylene and rehydrated using a graded series of ethanol. All slides were treated with NaBH4 to suppress autofluorescence of tissues. The expression levels of ArhGAP30, and SRSF3 were probed with the primary antibodies [ArhGAP30, dilution 1:50; SRSF3, dilution 1:100] according to the manufacturer’s instructions. Secondary antibodies (Alexa488-anti-rabbit) were used to label ArhGAP30 and SRSF3. After staining with DAPI (1:10,000), the coverslips were added with antifade reagent (ProLong Gold, Invitrogen) and kept in the dark for 24 h. Images were acquired with a confocal fluorescence microscope (Carl Zeiss).

### Cell lines and culture conditions

The human CRC cell lines, HCT116 and LoVo (ATCC, Manassas, VA, USA) were maintained in McCoy’5A, RPMI 1640 medium (Gibco, Gaithersburg, MD, USA) supplemented with 10% fetal bovine serum (Invitrogen) and cultured in a humidified incubator at 37 °C under 5% CO2.

### SRSF3 ShRNA construction

The shRNA sequence used to target SRSF3 was as follow: 5′-GGT TTATG TA GGCAAT CTTGG-3′; Synthetic oligonucleotide sequences were constructed, and annealed to create double-stranded DNA using the following sequences: forward, 5′-gatccGGTTTATGTAGGCAATCTTGGTTCAAGAGACCA AGATT GCCTACATAAACCTTTTTTg-3′; reverse, 5′-aattcAAAAAAGGTTTATGTAG GCAATC TTG GTCTCTTGAACCAAGATTGCCTACATAAACCg-3′. The target gene wasinserted into the AgeI and EcoRI cleaved pGC-LV vector by homologous recombination using the T4 DNA ligase enzyme. Competent DH5α cells were prepared with calcium chloride and subsequently transformed. The recombinant positive clones were sent to Genomeditech (Shanghai, China) for sequencing.

### SRSF3 plasmid construction and transfection

The pGMLV-SRSF3 plasmid containing the SRSF3 coding sequence was purchased from Shanghai Genomeditech (Shanghai, China) and verified by DNA sequencing. For plasmid transfection, HCT116 and LoVo cells were seeded into six-well plates 24 h before transfection, and then transfected with plasmids (4 mg per well) using Lipofectamine 2000 Reagent (Invitrogen), according to the manufacturer’s instructions. The pGMLV-vector was used as an empty vector control. After 48 h, cells were harvested for analysis.

### Western blot assay

Whole cell lysates were prepared from the cancer cell lines and standard Western blotting analysis was performed using anti-SRSF3(Abcam,USA), anti-ArhGAP30 (Abcam,USA), anti-p53(Santa Cruz,USA), anti-Acetylation p53(Epitomics, USA) and anti-GAPDH(Kangchen Biotechnology, Shanghai, China) antibodies. All primary antibodies were used at a 1:1000 dilution. Peroxidase-conjugated anti-goat or anti-rabbit IgG secondary antibodies were obtained from Kangchen Biotechnology and used at a 1:5000 dilution. Three independent experiments were done for each analysis.

### Cell viability assays

The stable transfected LoVo and HCT116 cells were seeded onto 96-well plates at 5000 cells/well. Cell proliferation was measured using the Cell Counting Kit-8 (CCK-8, Dojindo). At each time point, cells were incubated with 10µL CCK-8 reagent per well (100 µl medium/well) for 1 h at 37 °C, 5% CO2. The absorbance was measured at 450 nm. Data were presented as the percentage of viable cells as following:

$$ \text{Relative viability}\,= [\text{A450}\,(\text{treated}) {-} \text{A450}\,(\text{blank})]\,/\,[\text{A450}\,(\text{control}) {-} \text{A450}\,(\text{blank})]\,{ \times }\,{100\% }\,. $$

Three independent experiments were done for each analysis and recorded the same results.

### Soft agar colony formation assay

The HCT116 and LoVo cells (2*10^3^) treated with si-SRSF3 or control siRNA (p-SRSF3 or control plasmid) were plated into 24-well plates with 1% base agar and 0.5% top agar, and incubated for 2 (LoVo) or 3 weeks (HCT116). Colonies were fixed with 4% paraformaldehyde, and then stained with 0.5% crystal violet. Megascopic colonies were counted. The colonies were counted in eight randomly microscope fields. Three independent experiments were done for each analysis and recorded the same results.

### Cell invasion/migration assays

Cell invasion assays were performed using Boyden chambers with filter inserts (pore size, 8 µm) coated with 40µg Matrigel in 24-well plate dishes as described previously. Briefly, 2 × 105 LoVo or HCT116 cells stable transfected with shRNA-SRSF3, p-GMLV-SRSF3 or Control plasmid were seeded in the upper chamber, while the medium with 20% fetal bovine serum was placed in the lower chamber. The plates were incubated for 24 h. Then the cells were fixed in 4% formaldehyde and stained with 0.05% crystal violet in PBS for 20 min at room temperature. Cells on the upper side of the filters were removed by cotton-tipped swabs, and the filters were washed with PBS. The cells on the lower side of the filters were defined as invasive cells and counted at x200 magnification in 10 different fields of each filter. Three independent experiments were done for each analysis and recorded the same results.

### In vivo experiments

Briefly, male BALB/c athymic nude mice (4–5 weeks old) were obtained from the Experimental Animal Center of SIBS. Mice were randomly divided into two groups (8 mice/group): Control group and SRSF3 shRNA group. Control group mice were injected subcutaneously into the right armpit with 1.0 *10^7^ LoVo cells bearing empty plasmid vector, while the SRSF3 shRNA group mice were injected with 1.0 *10^7^ LoVo cells bearing SRSF3 shRNA plasmid to establish a CRC xenograft model. Tumor diameters were measured at regular intervals with digital calipers, and tumor volume was calculated by the formula: tumor volume (mm3) = shorter diameter2 × longer diameter/2. The tumor volume data are presented as mean ± SD (n = 8). After 3 weeks, all mice were sacrificed and subcutaneous tumors were collected for analysis. Our study was approved by the Animal Care and Use Committee of the Shanghai Jiao-Tong University School of Medicine Renji Hospital, Shanghai, China. All animal procedures were performed according to the guidelines developed by the China Council on Animal Care and the protocol approved by the Shanghai Jiao-Tong University School of Medicine, Renji Hospital, Shanghai, China.

### Statistical analysis

Data from at least three independent experiments were presented as the mean standard deviation (SD). Comparisons were performed using the Student’s paired *t* test, Spearman’s correlation test, or Chi square test; p < 0.05 was considered statistically significant.

## Results

### SRSF3 upregulation is prevalent in CRC and associates with poor prognosis

**Fig. 1:**
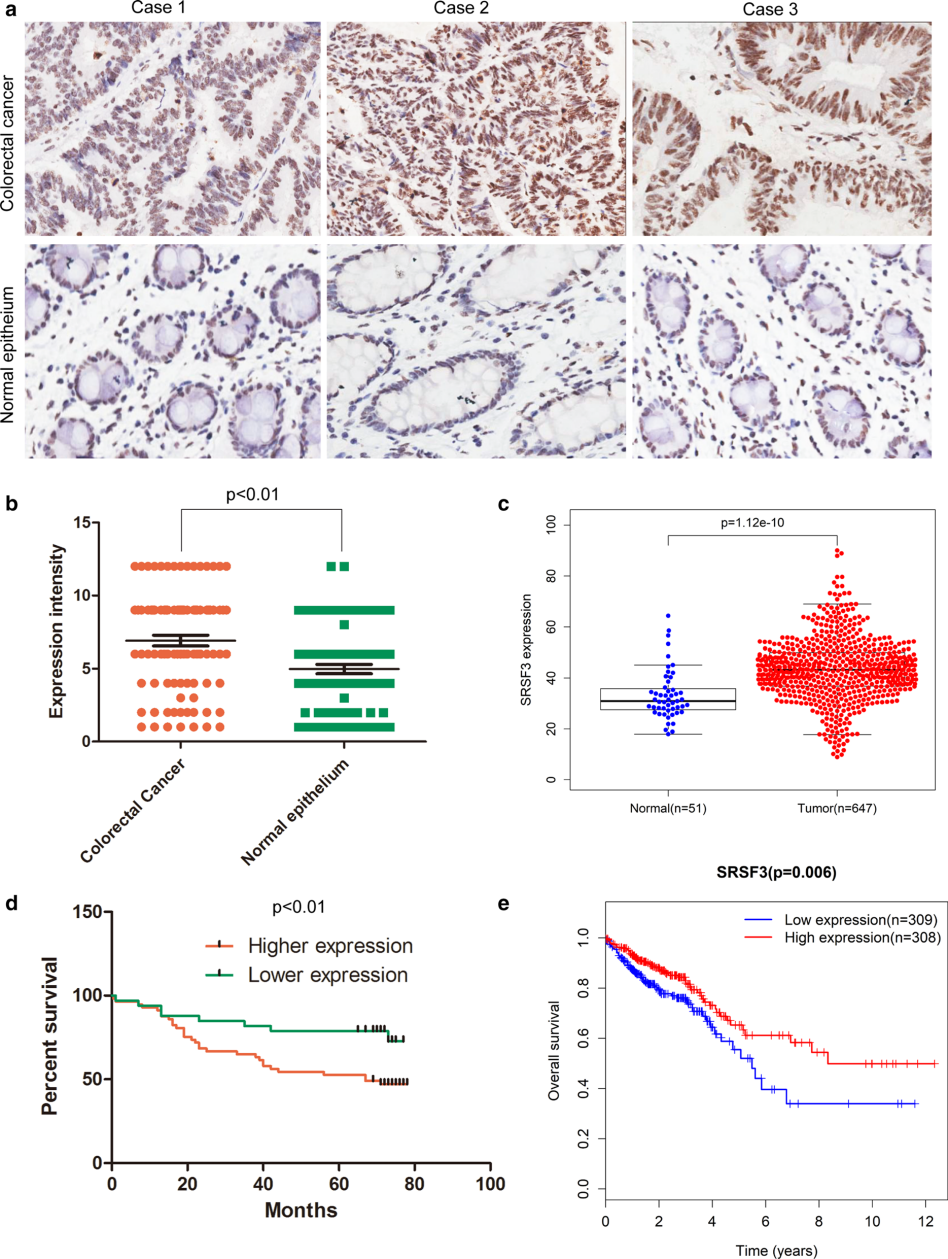
Correlation between ArhGAP30 expression and clinicopathological features of colorectal cancers: **a** Immunohistochemistry of SRSF3 in normal and CRC tissues; **b** Statistics of SRSF3 protein expression levels in normal and CRC tissues according to the immunohistochemistry analysis; **c** Statistics of ArhGAP30 protein expression levels in normal and CRC tissues based on the TCGA dataset; **d** Kaplan–Meier survival plot of patients stratified by SRSF3 protein expression level; **e** Kaplan–Meier survival analysis of an independent validation dataset from TCGA

First, by analyzing the expression of SRSF3 in CRC cases from 20 CRC cases and one tissue microarray including 90 CRC cases, we found significant higher levels of SRSF3 protein in CRC tissues than in normal colorectal tissues (p < 0.001, Fig. 1a, b). The higher expression of SRSF3 was also found in TCGA(the cancer genome atlas) CRC data (Fig. 1c). Next, by comparing different clinicopathological features of 90 CRC cases stratified by SRSF3 expression level, we found SRSF3 upregulation significantly associated with poorer differentiation (p = 0.01), more lymph node invasion (p = 0.01), and advanced AJCC stage (p = 0.01, all comparisons by Fisher’s exact test, Table 1). Upregulation of SRSF3 was also associated with shorter overall survival in our dataset (p < 0.01, Fig. 1d) and in TCGA CRC dataset (p = 0.006, Fig. 1e). Taken together, these results consistently demonstrated a tight association between SRSF3 upregulation and poor CRC prognosis, and suggested that SRSF3 may play a role in colorectal carcinogenesis.

**Table 1:** SRSF3 and clinicopathological features

	SRSF3 high	SRSF3 low	P value
Sex			
Male	30	17	
Female	27	16	0.92
Age			
< 60 years	17	11	
> 60 years	40	22	0.73
Location			
Left	30	14	
Right	27	19	0.35
Tumor size			
< 5 cm	25	14	
> 5 cm	32	19	0.89
Differentiation			
1,2	44	32	
3,4	13	1	0.01
T stage			
T1-2	9	4	
T3-4	48	29	0.63
Lymph node invasion			
N0	32	27	
N1	25	6	0.01
Distance metastasis			
M0	54	33	
M1	3	0	0.18
AJCC stage			
1,2	30	27	
3,4	27	6	0.01

### SRSF3 promotes the proliferation and invasion of CRC cells

**Fig. 2:**
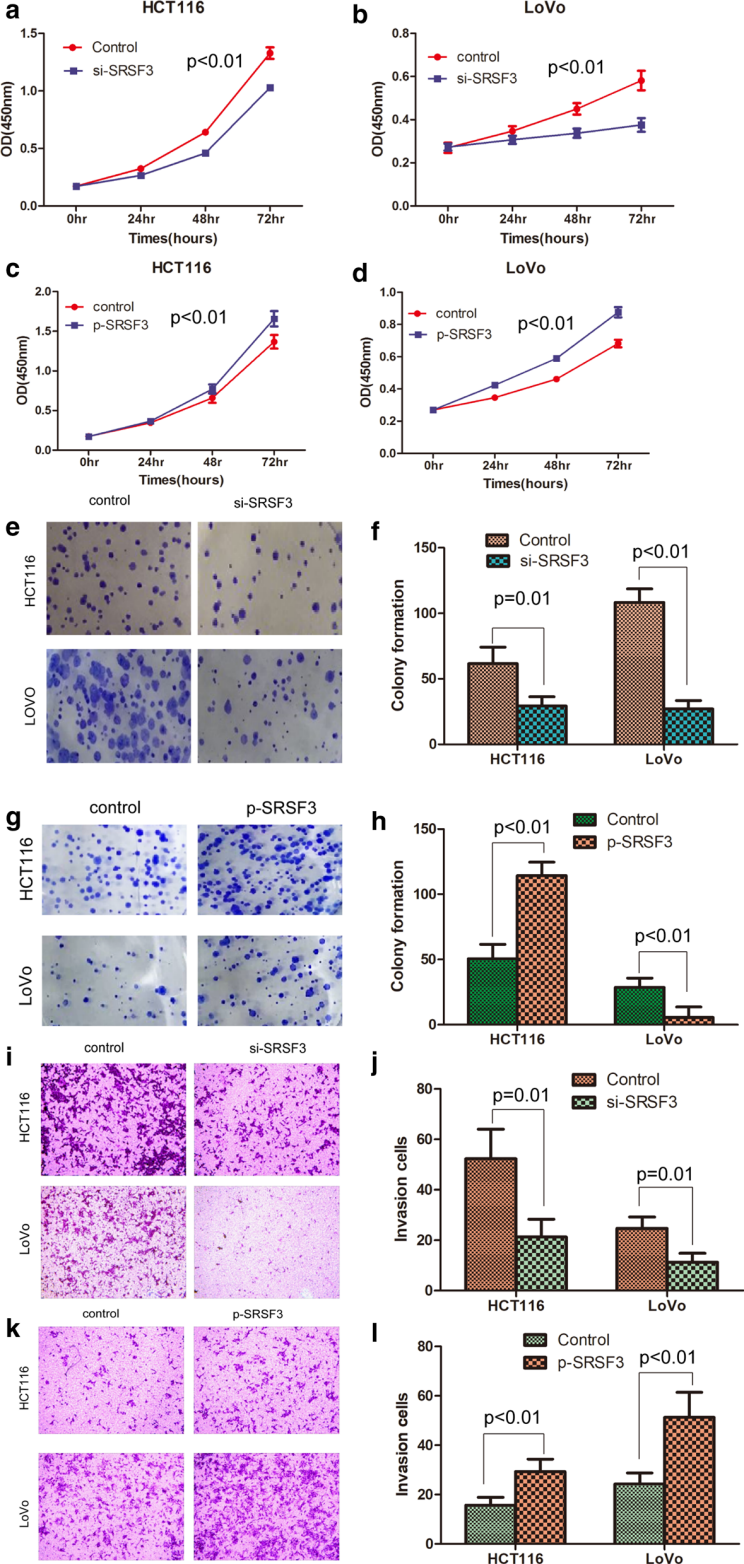
SRSF3 promotes the proliferation and invasion of CRC cells: **a**, **b** Proliferation curves of HCT116 (**a**) and LoVo cells (**b**) as determined by CCK-8 assay after knockdown of SRSF3 in cell lines; **c**, **d**. Proliferation curves of HCT116 (**c**) and LoVo cells (**d**) as determined by CCK-8 assay after ectopic expression of SRSF3 in cell lines; **e** Representative images of colony formation assay for HCT116 and LoVo cells after knockdown of SRSF3; **f** Statistics of colony formation assay for HCT116 and LoVo cells after knockdown of SRSF3; **g** Representative images of colony formation assay for HCT116 and LoVo cells after ectopic expression of SRSF3; H. Statistics of colony formation assay for HCT116 and LoVo cells after ectopic expression of SRSF3; **i** Representative images for Transwell invasion assay of HCT116 and LoVo cells after knockdown of SRSF3; J. Statistics of Transwell invasion assay of HCT116 and LoVo cells after knockdown of SRSF3; **k** Representative images for Transwell invasion assay of HCT116 and LoVo cells after ectopic expression of SRSF3; L. Statistics of Transwell invasion assay of HCT116 and LoVo cells after ectopic expression of SRSF3

In light of the above findings, we questioned whether SRSF3 functions as an oncogene in CRC. To confirm the effects of SRSF3 on cell proliferation and invasion, human CRC LoVo and HCT116 cells stably transfected with SRSF3 shRNA or p-GMLV plasmid and their corresponding control vector were analyzed by CCK-8, soft-agar colony formation assay and transwell invasion assays. Efficient knockdown of SRSF3 was confirmed in both mRNA and protein levels, ectopic expression of SRSF3 was also detected in both mRNA and protein levels (data not shown). As shown in Fig. 2, both CCK-8 and soft agar colony formation assay indicated that suppression of SRSF3 significantly decreased the proliferation rate of HCT116 and LoVo cells, while overexpression of SRSF3 significantly increased the proliferation rate of CRC cells. We further used transwell assay to monitor the effect of manipulating SRSF3 expression on cell invasiveness. Knockdown of SRSF3 significantly decreased the invasion rate of HCT116 and LoVo cells, while ectopic expression of SRSF3 significantly increased the proliferation rate of CRC cells.

### SRSF3 suppresses ArhGAP30/Acetylation-p53 in CRC cells

**Fig. 3:**
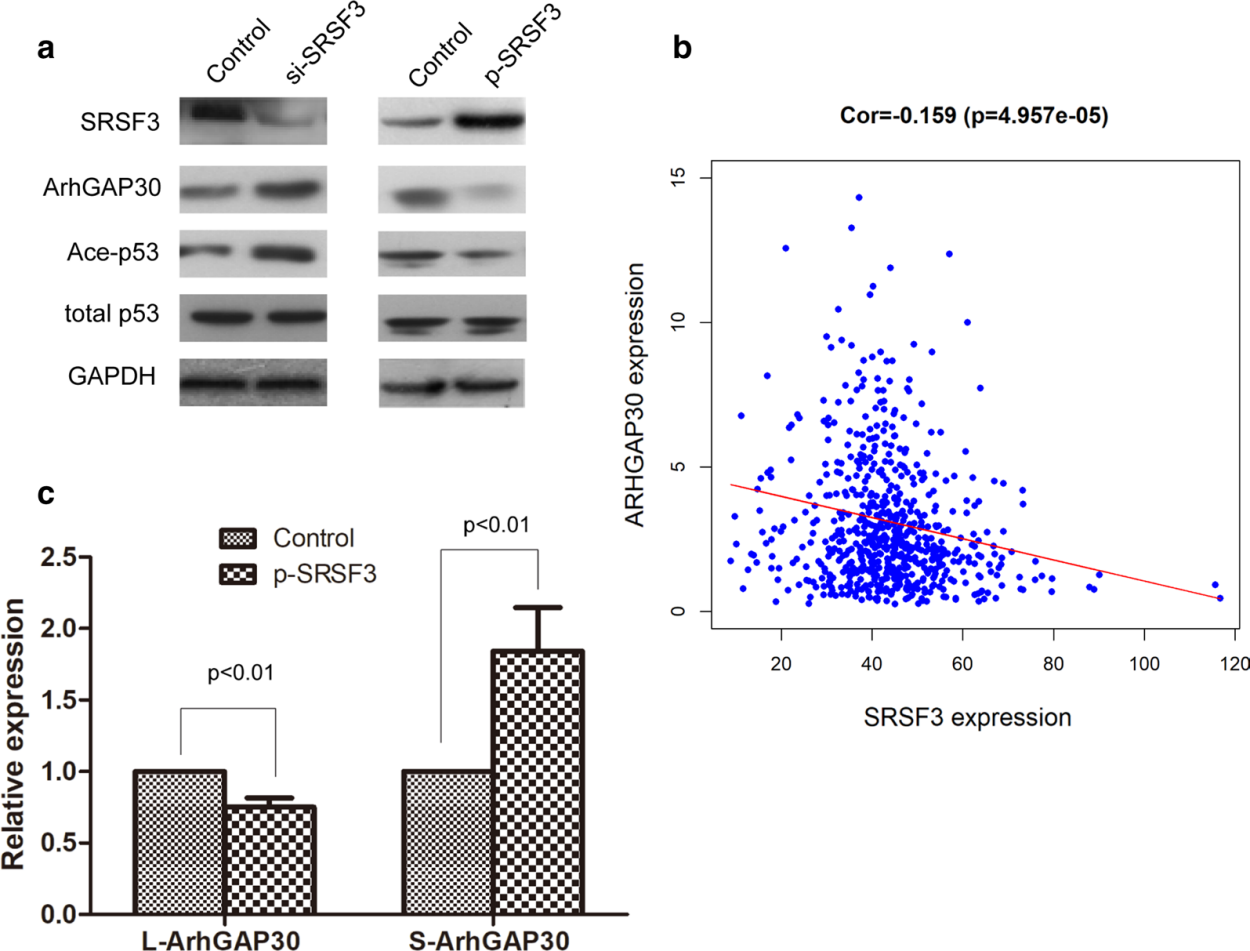
SRSF3 regulate the expression of ArhGAP30: **a** Western blot analysis shows that knockdown of SRSF3 significantly increase the expression of ArhGAP30 and Ace-p53, while overexpression of SRSF3 significantly decrease the expression of ArhGAP30 and Ace-p53; **b** Regression analysis reveals a reverse correlation between SRSF3 and ArhGAP30 in CRC tissues based on TCGA dataset; **c** Relative expression of L-ArhGAP30 and S-ArhGAP30 (two isoforms of ArhGAP30) after overexpression of SRSF3 in CRC cells

We next investigated the mechanism underlying the pro-malignancy effects of SRSF3. Since SRSF3 is a widely functioned alternative splicing factor, and ArhGAP30 has two splicing isoforms, L-ArhGAP30 and S-ArhgAP30, and the function of the two isoforms are quite different, therefore, we speculated that SRSF3 could regulate the expression and function of ArhGAP30. As expected, knockdown of SRSF3 could increase the expression of ArhGAP30 and Acetylation-p53, while ectopic expression of SRSF3 could suppress the expression of ArhGAP30 and Ace-p53 (Fig. 3a). Further analysis of TCGA CRC data revealed that SRSF3 expression was reversely correlated with ArhGAP30 expression in CRC tissues (Fig. 3b). Real time PCR found that ectopic expression of SRSF3 could decrease the expression of L-ArhGAP30 and increase the expression of S-ArhGAP30 (Fig. 3c), suggesting that SRSF3 may function by alternative splicing of ArhGAP30, however, the detailed mechanism need to be further explored. These data demonstrated that SRSF3 may function as an oncogene in CRC by suppresses ArhGAP30/Ace-p53 axis.

### Knockdown of SRSF3 suppresses xenograft growth in mice

**Fig. 4:**
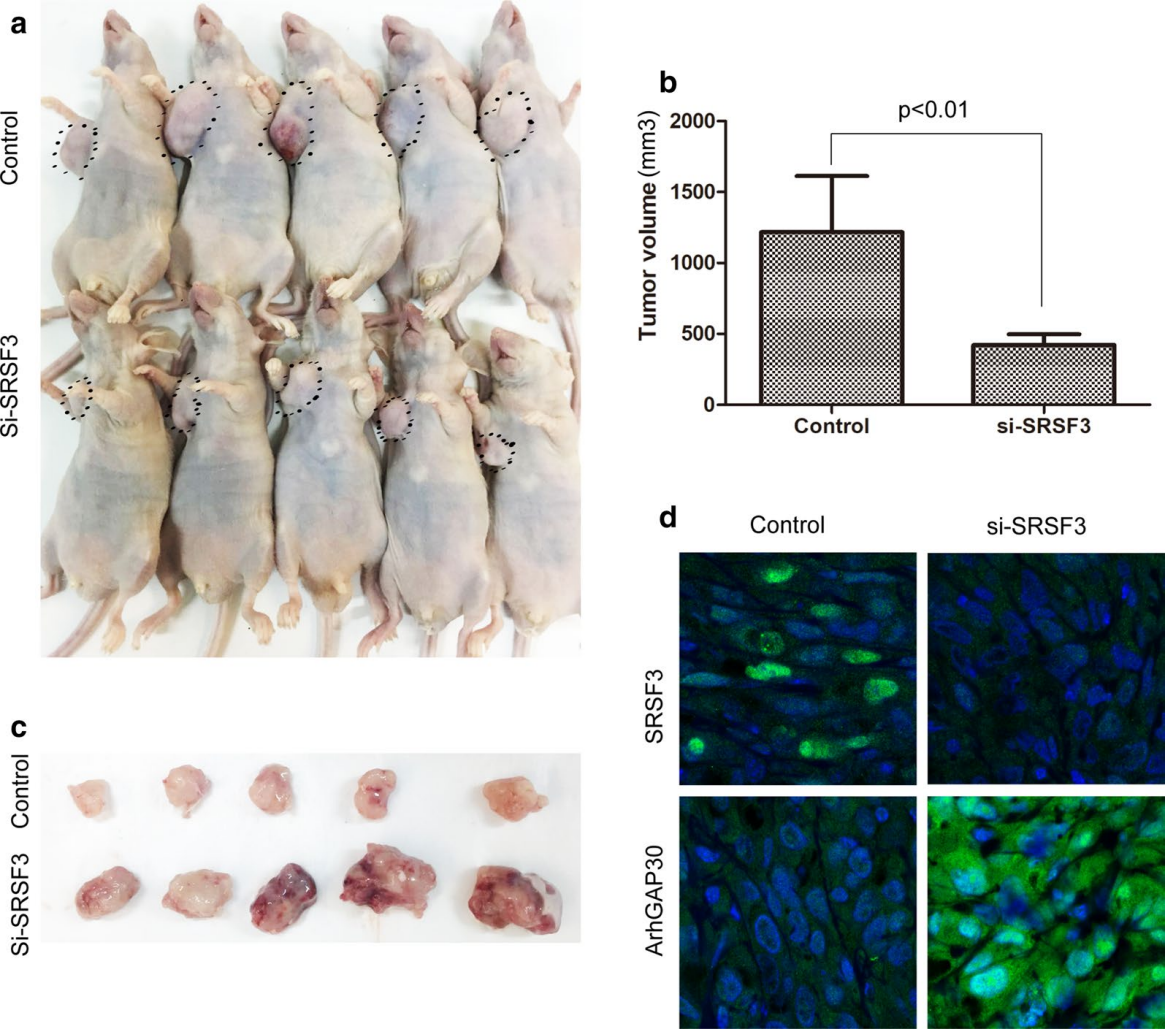
SRSF3 could serve as a potential therapeutic target in CRC: **a**, **b** Knockdown of SRSF3 inhibited growth of xenograft in nude mice; **c** Tumor volume in the SRSF3 siRNA group and the control group. Knockdown of SRSF3 significantly suppressed xenograft tumor progression. **d** Immunofluorescence showing knockdown of SRSF3 dramatically decreased the level of ArhGAP30 in xenograft CRC tumors

We found that knockdown of SRSF3 could suppress the growth of CRC cells in CCK-8 assay and soft agar colony formation assays. To further confirm the impact of SRSF3 on CRC growth in vivo, we established a LoVo xenograft tumor model in BALB/C nude mice. The human CRC LoVo cells bearing SRSF3 shRNA or control shRNA were separately implanted subcutaneously into nude mice to allow tumor formation. As shown in Fig. 4a–c, the tumor volume in the SRSF3 shRNA group was significantly smaller compared with the control group, indicating that SRSF3 knockdown could significantly suppresse CRC tumor growth in vivo. In accordance with these changes in phenotype, ArhGAP30 expression levels was significantly increased in the SRSF3 shRNA group (Fig. 4d). These data, together with the upregulation of SRSF3 in CRC, suggested that targeting SRSF3 may have therapeutic potential in CRC.

## Discussion

As one of the most important SR proteins, SRSF3 has got a lot of attentions, especially in cancer. Previous studies have revealed that SRSF3 was upregulated in many kinds of cancers, including CRC. Here, in consistent with these studies, we also found that SRSF3 was overexpressed in CRC tissues, and we further investigated the relationship between SRSF3 expression and clinical characteristics and prognosis of CRC patients. Our data could bring the prognostic significance of SRSF3 into attention.

Our data revealed SRSF3 as a promising biomarker for the diagnosis and prognosis of CRC. We found that SRSF3 was significantly upregulated in CRC tissues, and SRSF3 overexpression associated with poor survival in both our data and TCGA validation dataset. Moreover, ectopic expression of SRSF3 could significantly promote the proliferation and invasion ability of CRC cells, while knockdown of SRSF3 could suppress its proliferation and invasion ability. These data strongly suggested the oncogenic roles of SRSF3 in CRC. The CRC xenograft experiment in this study also revealed that suppressing SRSF3 could significantly inhibit tumor growth in vivo. All of these data supported the idea that SRSF3 could serve as a therapeutic target in CRC.

As a major alternative splicing factor, previous studies usually focused the alternative splicing roles of SRSF3 in carcinogenesis. Tang’s study [13] showed that SRSF3 could regulate cellular senescence through TP53 alternative splicing; Ajiro’s study [7] found that SRSF3 could regulate the expression of 60 genes and 20 miRNAs in human osteosarcoma cells by the global profiling of the SRSF3-regulated splicing events; Kuranaga’s study [10] indicated that SRSF3 as a PKM splicer played a positive role in cancer-specific energy metabolism; Gautrey’s study [14] identified SRSF3 could regulate the production of these functionally distinct HER2 splice variants and therefore maybe important for the regulation of HER2 signaling. In this study, we found that SRSF3 could regulate the expression of ArhGAP30, which has been identified as a pivotal regulator for p53 acetylation and functional activation in CRC [11]. Knockdown of SRSF3 could increase the expression of ArhGAP30, while ectopic expression of SRSF3 could decrease the expression of ArhGAP30. Moreover, overexpression of SRSF3 could decrease the L-ArhGAP30/S-ArhGAP30 ratio, thus resulting in loss of function of ArhGAP30 in CRC. These data suggested that SRSF3 could regulate the alternative splicing of ArhGAP30 in CRC, however, the detailed mechanism needs to be further clarified.

## Conclusion

In conclusion, our data suggested that increased SRSF3 expression could mediate CRC carcinogenesis and promote CRC progression by suppressing ArhGAP30 expression. Our results also highlight SRSF3 could serve as a promising biomarker and therapeutic target in CRC.

## Data Availability

All of the data and material could be available in Medline after publication.

## Abbreviations

CRC: Colorectal cancer
SR proteins: Serine/arginine-rich proteins
SRSF3: Serine/arginine-rich splicing factor 3
ArhGAP30: Rho GTPase activating protein 30
IHC: Immunohistochemistry
CCK-8: Cell counting Kit-8
TCGA: The cancer genome atlas

## References

[1] Bray F, Ferlay J, Soerjomataram I, Siegel RL, Torre LA, Jemal A (2018). Global cancer statistics 2018: GLOBOCAN estimates of incidence and mortality worldwide for 36 cancers in 185 countries. CA Cancer J Clin.

[2] Wang BD, Lee NH (2018). Aberrant RNA splicing in cancer and drug resistance. Cancers.

[3] Sveen A, Kilpinen S, Ruusulehto A, Lothe RA, Skotheim RI (2016). Aberrant RNA splicing in cancer; expression changes and driver mutations of splicing factor genes. Oncogene.

[4] Do DV, Strauss B, Cukuroglu E, Macaulay I, Wee KB, Hu TX (2018). SRSF3 maintains transcriptome integrity in oocytes by regulation of alternative splicing and transposable elements. Cell Discov.

[5] Jia R, Zhang S, Liu M, Zhang Y, Liu Y, Fan M (2016). HnRNP L is important for the expression of oncogene SRSF3 and oncogenic potential of oral squamous cell carcinoma cells. Sci Rep.

[6] Ke H, Zhao L, Zhang H, Feng X, Xu H, Hao J (2018). Loss of TDP43 inhibits progression of triple-negative breast cancer in coordination with SRSF3. Proc Natl Acad Sci USA.

[7] Ajiro M, Jia R, Yang Y, Zhu J, Zheng ZM (2016). A genome landscape of SRSF3- regulated splicing events and gene expression in human osteosarcoma U2OS cells. Nucleic Acids Res.

[8] Chang YL, Hsu YJ, Chen Y, Wang YW, Huang SM (2017). Theophylline exhibits anti-cancer activity via suppressing SRSF3 in cervical and breast cancer cell lines. Oncotarget.

[9] Lin JC, Lee YC, Tan TH, Liang YC, Chuang HC, Fann YC (2018). RBM4-SRSF3-MAP4K4 splicing cascade modulates the metastatic signature of colorectal cancer cell. Biochim Biophys Acta Mol Cell Res.

[10] Kuranaga Y, Sugito N, Shinohara H, Tsujino T, Taniguchi K, Komura K (2018). SRSF3, a splicer of the PKM gene, regulates cell growth and maintenance of cancer-specific energy metabolism in colon cancer cells. Int J Mol Sci.

[11] Wang J, Qian J, Hu Y, Kong X, Chen H, Shi Q (2014). ArhGAP30 promotes p53 acetylation and function in colorectal cancer. Nat Commun.

[12] Wang YC, Wang JL, Kong X, Sun TT, Chen HY, Hong J (2014). CD24 mediates gastric carcinogenesis and promotes gastric cancer progression via STAT3 activation. Apoptosis.

[13] Tang Y, Horikawa I, Ajiro M, Robles AI, Fujita K, Mondal AM (2013). Downregulation of splicing factor SRSF3 induces p53β, an alternatively spliced isoform of p53 that promotes cellular senescence. Oncogene.

[14] Gautrey H, Jackson C, Dittrich AL, Browell D, Lennard T, Tyson-Capper A (2015). SRSF3 and hnRNP H1 regulate a splicing hotspot of HER2 in breast cancer cells. RNA Biol.

